# Supplementary material for: The discovery of novel noncoding RNAs in 50 bacterial genomes
Source: Nucleic Acids Res. 2024 Apr 22;52(9):5152–65. doi: 10.1093/nar/gkae248 (PMC11109978; doi:10.1093/nar/gkae248)
Supplement: gkae248_Supplemental_Files [file gkae248_supplemental_files.zip › Narunsky 50 Genomes RNA Motifs Files.zip!/Publication/62-K_koreensis/Figures/LRC-62-29-NC_013166_1_IGR_78_1632409_1632791.sto.pdf]

LRC-62-29-NC_013166_1_IGR_78_1632409_1632791.sto.r2r

5′-GUUUGACUCCU●○AA●YUUAUR●GAGGCUY●AY○UCU●YUGUU●Y○ARCCUC●○RUY●AAAGY●●U●●GRCGAA●●○○●AYUA●CUUGUR●URAYRYYCGYCUAUUUAAGGUURGYCCRY●●Y●YU●AUAA●Y●GRYGAAA○●UYRCRGAC●●●GCGCA●G●A○●CAUACGCYA●○○AAYUGA○○○ACGCUAGCUCCAYUGAGCGCGAAUYUGGUCGGCAACGAUAAACGUUGGUGUGAUUUGUUUR●URAAYAUCAAUAACAUAUA●●YU●UUUGCCCUGUAACAGCUUGAUUUUAAAGURGCUA●UUACGAUCCCY●CAUUAUGCAACAGCCCGCYAAUAUUAACAAUAUCGCGGCUGCUU●R○●UAGAA●GUUCAGYUAUR○
